# Novel CT-based classification of Hoffa fracture and optimal treatment strategies

**DOI:** 10.3389/fsurg.2025.1564933

**Published:** 2025-05-26

**Authors:** Haiming Chen, Mingwei He, Guodong Li, Jinqiu Wang, Kaimin Yang, Qingjun Wei

**Affiliations:** ^1^ Department of Orthopedics Trauma and Hand Surgery, The First Affiliated Hospital of Guangxi Medical University, Nanning, Guangxi, China; ^2^ Department of Orthopedics, The First People’s Hospital of Yulin, Yulin, Guangxi, China; ^3^ Department of Orthopedics, Minzu Hospital of Guangxi Zhuang Autonomous Region, Nanning, Guangxi, China; ^4^ Physical Examination Center, Red Cross Hospital of Yulin City, Yulin, Guangxi, China; ^5^ Department of Orthopedics, The Second Affiliated Hospital of Guangxi Medical University, Nanning, Guangxi, China

**Keywords:** Hoffa fracture, classification, optimal treatment strategies, retrospective analysis, CT imaging characteristics

## Abstract

**Background:**

The imaging characteristics of Hoffa fracture are gradually changing, leading to limitations of the existing classification system and necessitating the development of a novel classification system to guide clinical management.

**Objective:**

This study proposes a novel classification method for Hoffa fracture through a retrospective analysis of CT imaging characteristics, biomechanical studies, and case reports and suggests corresponding surgical approaches and internal fixation methods.

**Method:**

The CT imaging characteristics of 115 adults with Hoffa fractures from five tertiary hospitals were analyzed, accompanied by a retrospective review of biomechanical studies and case reports, to propose a novel classification system. Corresponding surgical approaches and internal fixation methods were recommended. Six independent observers evaluated the inter- and intra-observer reliability of the novel classification system.

**Results:**

The new classification method includes four broad types, and the recommended surgical approach and fixation strategy for each type have been identified. The mean Kappa coefficients for the first and second rounds of inter-observer were 0.766 and 0.752, respectively, with a mean Kappa coefficient for intra-observer of 0.853.

**Conclusion:**

The proposed classification system aligns closely with the clinical characteristics of Hoffa fracture, facilitates the differentiation of various fracture types, and recommends appropriate surgical approaches and fixation methods. This classification method also presents good inter- and intra-observer reliability and can be used in clinical management.

## 1 Introduction

Hoffa fracture is a coronal fracture of the femoral condyle that was first described systematically by Hoffa in 1904 (1). This type of fracture is relatively rare, with unicondylar Hoffa fractures constituting approximately 0.6% of all femoral fractures (2). Multiple classification systems are available for Hoffa fracture, and the Letenneur classification is the most widely utilized. Despite its prevalence, this classification has certain limitations. It does not encompass all types of Hoffa fractures and lacks guidance concerning surgical approaches and internal fixation methods, thereby diminishing its clinical utility. The present study proposes a novel classification system based on summarizing the CT imaging characteristics and previous biomechanical studies. It retrospectively analyzes surgical approaches and internal fixation methods in case reports to identify corresponding surgical approaches and internal fixation methods for each type.

## 2 Materials and methods

### 2.1 Case study

We conducted a retrospective analysis of clinical data from 115 adult patients with Hoffa fractures admitted to the First, Second, and Sixth Affiliated Hospitals of Guangxi Medical University, the Nationalities Hospital of Guangxi Zhuang Autonomous Region, and the Red Cross Hospital of Yulin between July 2014 and June 2024. The inclusion criteria included diagnosis of distal femoral Hoffa fracture among hospitalized patients, presence of comprehensive clinical data, age ≥18 years old, and clear morphology of fracture fragments. The exclusion criteria included the following: patients who were followed up, old fractures, pathological fractures, severely comminuted fractures that cannot be staged, and Hoffa fracture surgery conducted in other hospitals.

Data were analyzed using IBM SPSS 26.0 software. Quantitative data are presented as mean ± standard deviation, while categorical data are described using frequency and percentage. Descriptive statistics were employed to characterize the imaging features of Hoffa fractures.

### 2.2 Literature search

1) Relevant literature was searched using terms such as “Hoffa fracture”, “femoral condyle coronal fracture”, “biomechanics”, and “mechanics” in PubMed and China National Knowledge Infrastructure (CNKI). The inclusion criteria included the following: articles of CNKI sourced from core journals; distal femoral Hoffa fracture; cadaver specimens or artificially synthesized specimens; and use of English language. Finite element mechanics analyses, duplicate articles, and those not related to the biomechanical study of Hoffa fracture were excluded.
2) Relevant literature was searched using terms such as “Hoffa fracture”, “femoral condyle coronal fracture”, “Osteochondral fracture of the distal femur”, “Letenneur classification”, and “33-B3” in PubMed and CNKI. The inclusion criteria were CNKI article sourced from core journals; written in English. reports or series regarding Hoffa fracture; literature discussing the surgical treatment of Hoffa fracture; age ≥18 years old; exact Letenneur types; detailed case numbers and corresponding surgical approaches and fixation methods for each type; and fresh fracture. The exclusion criteria included the following: old fracture; nonunion fracture; pathological fracture; inability to obtain the surgical approaches and fixation methods for each type; animal model studies; literature reviews; and duplicate articles.

We analyzed fracture types, surgical approaches, and internal fixation methods in both literature sections.

### 2.3 Propose a novel classification

We propose a novel classification system that categorizes Hoffa fractures into four types (I–IV), building upon the Letenneur classification framework while integrating radiological characteristics of these fractures with biomechanical evidence and clinical insights from existing case studies. This classification system is determined by the size of the fracture fragment and whether it is unicondylar or bicondylar. Types I, II, and III are further sub-classified into Ia, Ib, IIa, IIb, IIIa, and IIIb based on whether the fracture is comminuted, or the articular surface is collapsed. Finally, 0 and 1 describe whether these subtypes are combined with intercondylar and/or supracondylar fractures.

### 2.4 Inter-observer and intra-observer variability test

Ten cases were randomly selected and classified by six orthopedic surgeons of varying experience levels (two junior doctors, two intermediate doctors, and two senior doctors) according to the novel classification system based on CT imaging data. Subsequently, we assessed inter- and intra-observer variations and repeated the above procedure three weeks later. Data from each observer following the complete protocol were included in the statistical analysis. Statistical analyses were performed using IBM SPSS Statistics 26.0. Inter-observer consistency among the six raters was assessed using Fleiss's Kappa coefficient, while intra-observer consistency was assessed with Cohen's Kappa coefficient, both reported with 95% confidence intervals. The values of kappa were interpreted according to the guidelines of Landis and Koch (3).

## 3 Results

### 3.1 Radiological characteristics of Hoffa fracture

A total of 115 distal femoral Hoffa fractures, involving 116 knees and 135 condyles, met the study criteria. The imaging characteristics of these fractures are presented in Table 1.

**Table 1:** Imaging characteristics of Hoffa fractures.

Characteristics	No. of cases (ratio)	Characteristics	No. of cases (ratio)
Age (years)	48.50 ± 15.56	Open fracture	
Gender		Yes	45 (38.79%)
Male	71 (61.74%)	No	71 (61.21%)
Female	44 (38.26%)	Femoral condyle	
Knee		Isolated medial condyle	22 (18.97%)
Both	1 (0.87%)	Isolated lateral condyle	20 (17.24%)
Right	59 (51.30%)	Non-isolated unicondyle	55 (47.41%)
Left	55 (47.83%)	Bicondyle	19 (16.38%)
Combined with intercondylar and/or supracondylar fractures		Letenneur classification	135 condyles
Yes	78 (67.24%)	I	63 (46.67%)
No	38 (32.76%)	II	22 (16.30%)
Comminuted fracture		III	48 (35.56%)
Yes	70 (60.35%)	Unspecified	2 (1.48%)
No	46 (39.66%)		

### 3.2 Biomechanics research

Of the 92 articles searched, 8 met the research criteria. The Letenneur classification and corresponding internal fixation methods recommended are presented in Table 2.

**Table 2:** Biomechanical studies of Hoffa fractures.

No.	Letenneur classification	Internal fixation implement	Recommended internal fixation methods	Literature
1	I, II	Fully threaded cortical screws (4.5 mm)	Type I: AP or PA Type II: PA	Peez et al. (4)
2	I	AO cannulated lag screws (7.3 mm), Acutrak headless compression screws (7.0 mm)	Acutrak headless compression screws, AP or PA	Peng et al. (5)
3	I	Partially threaded cannulated screws (6.5 mm)	AP or PA	Yao et al. (6)
4	I	Partially threaded cancellous screws (6.5 mm)	PA	Jarit et al. (7)
5	II	Two screws	Type IIa: AP or PA Type IIb, IIc: PA	Liu et al. (8)
6	I	Partially threaded cannulated screws (6.5 mm), LCP metaphyseal plate (3.5 mm)	PA screws plus a lateral LCP metaphyseal plate	Sun et al. (9)
7	I	Cannulated lag screws (3.5 mm), Buttressing locking plate, One-third tubular locking plate	PPA screws plus a posterolateral buttressing locking plate	Pires et al. (10)
8	II	Cortical screws (3.5 mm), Partially threaded cancellous screws (6.5 mm)	AP	Hak et al. (11)

AP, anterior to posterior; PA, posterior to anterior; LCP, locking compression plate.

### 3.3 Case reports

From the 471 articles retrieved, we selected 56 studies for further analysis, encompassing data from 267 patients. The surgical approaches and internal fixation methods are as follows. (1) Types I and III frequently employ the lateral approach, where the screws are inserted from anterior to posterior. (2) Type II is frequently managed by a posterolateral or posteromedial approach, where the screws are inserted from posterior to anterior. (3) Bicondylar Hoffa fractures are typically managed through the Swashbuckler approach, with a plate in conjunction with screws for internal fixation. (4) In cases of osteochondral fracture, a parapatellar, medial, or anterior approach may be used, where screws are inserted from posterior to anterior. (5) For the anterior Hoffa fracture, the parapatellar approach is typically employed, in which screws are inserted from anterior to posterior (Tables 3, 4).

**Table 3:** Surgical approaches for various fracture types.

Surgical approach	Unicondyle			Bicondyle	Osteochondral fracture	Anterior Hoffa fracture
	I	II	III			
Medial or lateral approach	5	8	4		1	
Posterolateral or posteromedial approach	30	23	18			
Parapatellar approach	69	19	53	1	1	1
Extend Carlson approach	8	6	3			
Subvastus approach	2					
Swashbuckler approach		1		3		
Arthroscopic approach	1	2	3			
Posterior approach		2				
Medial and lateral approach				1		
Gerdy's tubercle osteotomy approach				1		
Patella osteotomy approach				1		

**Table 4:** Internal fixation methods for various fracture types.

Internal fixation methods		Unicondyle			Bicondyle	Osteochondral fracture	Anterior Hoffa fracture
		I	II	III			
Screws	Anterior to posterior	47	13	37	2		1
	Posterior to anterior	18	28	16		2	
	Other configuration		1		1		
Plates with screws		42	16	25	3		
Plates alone					1		
Unspecified		8	3	3			

### 3.4 Novel classification system for Hoffa fractures

The classification system is predominantly based on fracture characteristics observable in CT images.

Type I: The fracture fragment encompasses the entire posterior condyle of the femur, where the primary fracture line is parallel to the posterior femoral cortex or slopes anteriorly, equivalent to the Letenneur types I and III. The subtypes are as follows:
  Ia-0 refers to simple splitting fracture, not combined with intercondylar and/or supracondylar fractures;
  Ia-1 refers to simple splitting fracture combined with intercondylar and/or supracondylar fractures;
  Ib-0 refers to comminuted fracture or collapsed articular surface, not combined with intercondylar and/or supracondylar fractures;
  Ib-1 refers to comminuted fractures or collapsed articular surface, combined with intercondylar and/or supracondylar fractures.
Type II: The fracture fragment constitutes a portion of the posterior condyle of the femur. The principal fracture line lies posterior to the posterior femoral cortex, equivalent to the Letenneur type II. The subtypes are as follows:
  IIa-0 refers to simple splitting fracture, not combined with intercondylar and/or supracondylar fractures;
  IIa-1 refers to simple splitting fracture combines with intercondylar and/or supracondylar fractures;
  IIb-0 refers to comminuted fracture or articular surface collapse, not combined with intercondylar and/or supracondylar fractures;
  IIb-1 Comminuted fracture or articular surface collapse, combined with intercondylar and/or supracondylar fractures.
Type III: Bicondylar Hoffa fracture. The subtypes are as follows:
  IIIa-0 refers to simple splitting fracture, not combined with intercondylar and/or supracondylar fractures;
  IIIa-1 refers to simple splitting fracture combines with intercondylar and/or supracondylar fractures;
  IIIb-0 refers to comminuted fracture or articular surface collapse, not combined with intercondylar and/or supracondylar fractures;
  IIIb-1 refers to comminuted fracture or articular surface collapse, combined with intercondylar and/or supracondylar fractures.
Type IV: Special types include anterior Hoffa and osteochondral fractures.

According to the novel classification system, the fracture types of 116 knees are presented in Table 5. Type Ib-1 is the most common.

**Table 5:** Fracture types of cases based on the novel classification system.

Classification	No. of cases	Classification	No. of cases
Ia-0	16 (13.79%)	IIIa-0	2 (1.72%)
Ia-1	19 (16.38%)	IIIa-1	1 (0.86%)
Ib-0	5 (4.31%)	IIIb-0	2 (1.72%)
Ib-1	38 (32.76%)	IIIb-1	14 (12.07%)
IIa-0	4 (3.45%)	IV	2 (1.72%)
IIa-1	2 (1.72%)		
IIb-0	8 (6.70%)		
IIb-1	3 (2.59%)		

### 3.5 Inter-observer and intra-observer correlation

All observers demonstrated full compliance with the aforementioned protocol throughout the study execution. The Kappa values are 0.774 for the first round of inter-observer and 0.782 for the second round. The mean Kappa value for intra-observer is 0.844 ± 0.057 (ranging from 0.770 to 0.885) (Table 6).

**Table 6:** The kappa values for intra-observer agreement.

Intra-observer	1-1′	2-2′	3-3′	4-4′	5-5′	6-6′	Mean
Kappa value	0.770	0.880	0.885	0.878	0.882	0.770	0.844 ± 0.057

## 4 Discussion

Hoffa fracture, a rare fracture involving the knee joint, constitutes 8.7%–13% of distal femoral fractures (12). The injury mechanism of Hoffa fracture remains unclear and is frequently associated with high-energy trauma, such as traffic accidents, fall injury from height, and crush injuries (13, 14). Hoffa fractures can occur not only through bone but also via the thick cartilage layer in young patients (15). The fracture line of Hoffa fracture is complex and varies significantly, with various treatment options available and varying prognoses. In our study, Hoffa fractures exhibit three distinct characteristics: a high prevalence of open fractures, a high prevalence of comminuted fractures, and a high prevalence of intercondylar and/or supracondylar fractures. Hence, most Hoffa fractures are caused by high-energy trauma and present complex patterns.

The fundamental imaging examination for Hoffa fracture is anteroposterior and lateral x-rays. Nevertheless, owing to the overlap of bone in the anteroposterior and lateral films, the interpretation of images becomes challenging, and the fracture is frequently missed. Only 69% of coronal fractures can be accurately diagnosed on x-rays (16). Thus, oblique and stress views should be added for assessment (17, 18). We contend that x-rays, which merely offer 2D images, possess restricted diagnostic value in light of the knee joint's complex anatomy and Hoffa fracture's multiplicity morphology. In addition, the passive position makes it difficult to achieve standard x-ray films. As our study showed, CT offers a 3D view of the fracture site, distal femur, and proximal tibia and is highly valuable for fracture assessment. Currently, it is considered the gold standard for diagnosing Hoffa fracture (19). An MRI examination is needed to comprehensively assess knee injuries, especially those concerning ligaments, menisci, and soft tissues (20). Moreover, in pediatric patients, considering that some fracture fragments might not be detectable on x-rays and a risk of radiation exposure exists. De Beer et al. recommended MRI as the preferred examination (15).

Numerous classification systems for Hoffa fractures have been put forward by scholars at home and abroad, encompassing the AO classification system and its modified forms, the Letenneur classification system and its modified variations, and the CT-based classifications established by Li et al., Bagaria et al., Sun et al., and Chandrabose et al. separately. Each of these classification systems has its advantages and disadvantages. The most classic and commonly used classification is the Letenneur classification. Letenneur (21) proposed this classification in 1978, which is based on plain radiographs. However, given the inherent limitations of plain radiography in fracture assessment and insufficient consideration of comminuted fractures along with those involving both intercondylar and supracondylar regions, we contend that the Letenneur classification fails to meet contemporary clinical requirements. The AO classification offers a unified categorization for diverse types of fractures throughout the entire body, among which Hoffa fractures are classified as type 33B3.2 (unicondyle fracture) and type 33B3.3 (bicondylar fracture) (22). However, this system is deficient in a detailed account of Hoffa fracture's specific morphology, making it difficult for clinicians to obtain valuable information. The AO-modified classification system further refined the classification of Hoffa fractures; nevertheless, it was deficient in describing the fragmentation and collapse of the joint surface and fails to encompass rare fracture types. Moreover, it fails to delineate bicondylar fractures combined with supracondylar and/or intercondylar fractures. In our research, comminuted fractures constituted 60.35%, and bilateral condylar fractures combined with supracondylar and/or intercondylar fractures made up 12.93%, representing an undeniable characteristic.

With the wide application of CT, many scholars have proposed CT-based classifications. Bagaria et al. (23) proposed a classification system based on CT images, encompassing the status of fracture fragments, degree of comminution, etc. The threshold for the size of fracture fragments was defined as 2.5 cm. This comprehensive classification encompasses rare fracture types and has greater guiding significance. Nevertheless, the threshold value is ascertained based on the characteristics of partial threaded screws. For the fixation of Hoffa fractures, multiple types of screws are as follows: cortical screws, cancellous screws 2 double-headed compression screws, fully threaded compression screws, and headless compression screws. Owing to the variations in body shape and size across age and gender, the size of the femoral condyle differs among individuals. Even within the same Letenneur type, individual differences can lead to variations in size. It is inherently deficient to employ a fixed numerical value as a threshold. Subtypes within type IV internal fractures exhibit significant variations. These encompass the relatively common bicondylar fractures and those involving supracondylar, as well as rare cases of anterior Hoffa fracture and osteochondral fracture, thereby making it challenging to master and guide surgical strategies. In the CT-based classification system proposed by Chandrabose et al. (24), internal instability was found to be triggered by the comminution of the cortex at the proximal of the fracture in the posterior area. Hence, in their classification, they emphasized the comminution of the fracture and its location. However, this classification fails to distinguish the sizes of the fracture fragments, and the size exerts a considerable influence on the surgical approach, selection of internal fixation devices, and fixation methods (25). Intercondylar and supracondylar fractures are excluded by this classification. Numerous classification methods show a deficiency of attention to intercondylar and supracondylar fractures. In our study, the proportion of cases combined with intercondylar and/or supracondylar fractures was as high as 67.24%, while the research conducted by Nork et al. also discovered that the incidence of coronal fractures accompanying intercondylar and supracondylar fractures was 38% (16), and Richards et al. (26) found that the proportion was reached as high as 52.7%, which constitutes a highly significant imaging feature. The medial and lateral condyles constitute important anatomical structures of the distal femur, and fractures in the intercondylar and supracondylar regions notably influence the fixation strategy for Hoffa fractures. The four anatomical structures are closely related. Referring to the corresponding tibial plateau, Hoffa fractures should be treated as a whole, along with intercondylar and supracondylar fractures. The latest classification system was proposed by Pires et al. (27) in 2022; however, his research solely focused on the classification of isolated medial fractures. As the number of high-energy injuries increases, the fracture morphology becomes progressively complex, with occasional occurrences of anterior Hoffa and osteochondral fractures, as noted by Bagaria et al. (23). De Beer et al. (15) contend that the Letenneur and AO classifications cannot be applied to osteochondral fractures. Two anterior Hoffa fractures were identified in our cohort for which the Letenneur classification proved inapplicable.

The need for classification arose to describe the severity of fractures, the increased requirement of surgical skill and expertise, the type of fixation preferred, and the prediction of the final prognostic outcome (24). An ideal classification system is expected to encompass all types and subtypes of fractures, possess universal applicability, be readily comprehensible, be reproducible and reliable across various modalities and among different levels of users, and exert a role in guiding treatment decisions and revealing prognosis (23, 28). Based on the Letenneur classification and incorporating the radiological characteristics of Hoffa fractures as well as insights from previous biomechanical studies and case reports, Hoffa fracture have been categorized into four types (I–IV). This classification is determined by the size of the fracture fragment and whether it is unicondylar or bicondylar. Then, based on whether the fracture is comminuted or if there is a collapse of the articular surface, types I, II, and III are further subdivided into subtypes a and b. Finally, 0 and 1 describe whether these subtypes are combined with intercondylar and/or supracondylar fractures.

Before proposing the novel classification system and corresponding treatment strategies, we comprehensively analyzed the differences between each type of Hoffa fracture in terms of anatomical structures, biomechanics, imaging, and previous treatment methods. Letenneur type I and III Hoffa fractures exhibit relatively large fragments involving the entire posterior femoral condyle with intact surrounding soft tissues, collectively accounting for 82.23% in our study. In contrast, Letenneur type II Hoffa fractures exhibit smaller fragments, present as complete intra-articular fractures with a lack of soft tissue attachment and poor blood supply, showing a lower proportion (16.30%). Significant differences exist between Letenneur type I/III and type II Hoffa fractures in anatomical and imaging features. Regarding the biomechanics of Hoffa fractures, recent studies suggest comparable mechanical performance between AP and PA screws in Letenneur type I fractures (4, 6), whereas Liu et al. (8) emphasizes that AP screws provide enhanced stability with larger fragments. For Letenneur type II fractures, both Liu et al. (8) and Peez et al. (4) demonstrated the biomechanical superiority of PA screws. In clinical case reports, Letenneur types I and III are predominantly treated via anterior approaches with AP screw fixation, while Letenneur type II is managed through posterior approaches using PA screws. Consequently, we categorize Letenneur types I and III as a unified group recommending AP screw fixation and classify Letenneur type II as a distinct category with PA screw fixation. To distinguish from the unicondylar Hoffa fracture, we categorize the bicondylar Hoffa fracture as the new classification type III. Rare types are classified as the new classification type IV. The four new classification types feature significant variances in anatomical structure, biomechanics, imaging, and treatment approaches. These distinctions can facilitate better discrimination and comprehension and are more consistent with clinical scenarios.

In our novel classification, type I is subdivided into four subtypes: Ia-0, Ia-1, Ib-0, and Ib-1. For Type I, the medial parapatellar approach (MPPA) or Medial subvastus approach (MSVA) for the medial condyle, the lateral parapatellar approach (LPPA) approach for the lateral condyle, and the Swashbuckler approach for certain complex fractures such as type Ia-1 and Ib-1. Concerning the selection of the internal fixation method, based on our previous analysis, we uniformly recommend adopting the anterior to posterior direction for screw insertion. We recommend inserting two compression screws for type Ia-0 and adding additional transverse screws or lateral locking plate for type Ia-1. Type Ib-0 is fixed with two cortical screws. Subsequently, depending on the extent of comminution or collapse at the fracture plane or joint surface, either bone grafting is chosen, and the medial/lateral plate with pinning technique is employed for fixation (29), or the posteromedial/posterolateral plate is utilized for fixation. Type Ib-1 is fixed with a medial/lateral locking plate in conjunction with screws, and the use of the pinning technique might be requisite to sustain the flatness of the joint surface.

Type II is also further classified into Ia-0, Ia-1, Ib-0, and Ib-1. In the cases of Type IIa-0 and IIb-0, the medial condyle is accessed through direct medial approach (DMA) or posteromedial approach, whereas the lateral condyle is accessed using direct lateral approach (DLA) or posterolateral approach; for Type IIa-1, the PPA approach or posteromedial/posterolateral approach is employed, and for Type IIb-1, the PPA approach is utilized, and MSVA is also an alternative for medial condyle. The main difference between Type II and I fracture fixations lies in the orientation of the screws. Biomechanical investigations by Peez et al. (4) and Liu et al. (8) have demonstrated the mechanical advantages of posterior to anterior screw fixation in stabilizing small fracture fragments. Meanwhile, this technique has been adopted by most surgeons in clinical practice. We suggest that all screws be inserted posterior to anterior. We propose to insert two compression screws for type IIa-0 and to add additional transverse screws or lateral locking plate for type IIa-1.Type IIb-0 is fixed with two cortical screws. Additionally, bone grafting is selected depending on the degree of comminution or collapse at the fracture plane or joint surface, and the medial/lateral plate with pinning technique is utilized for fixation (29). If the fracture fragment is tiny, then it might require a combination with a posteromedial/posterolateral plate. Type IIb-1 is fixed with a medial/lateral locking plate in combination with screws, and the application of the pinning technique might be necessary to maintain the flatness of the joint surface.

Type III refers to bicondylar Hoffa fracture, divided into four subtypes: IIIa-0, IIIa-1, IIIb-0, and IIIb-1. Adopting the Swashbuckler approach or a combined lateral and medial approach is advisable. According to the size and shape of the medial and lateral fracture fragments, and referencing the new classification Types I and II, the fixation is carried out either by using screws alone or in combination with locking plates.

**Figure 1:**
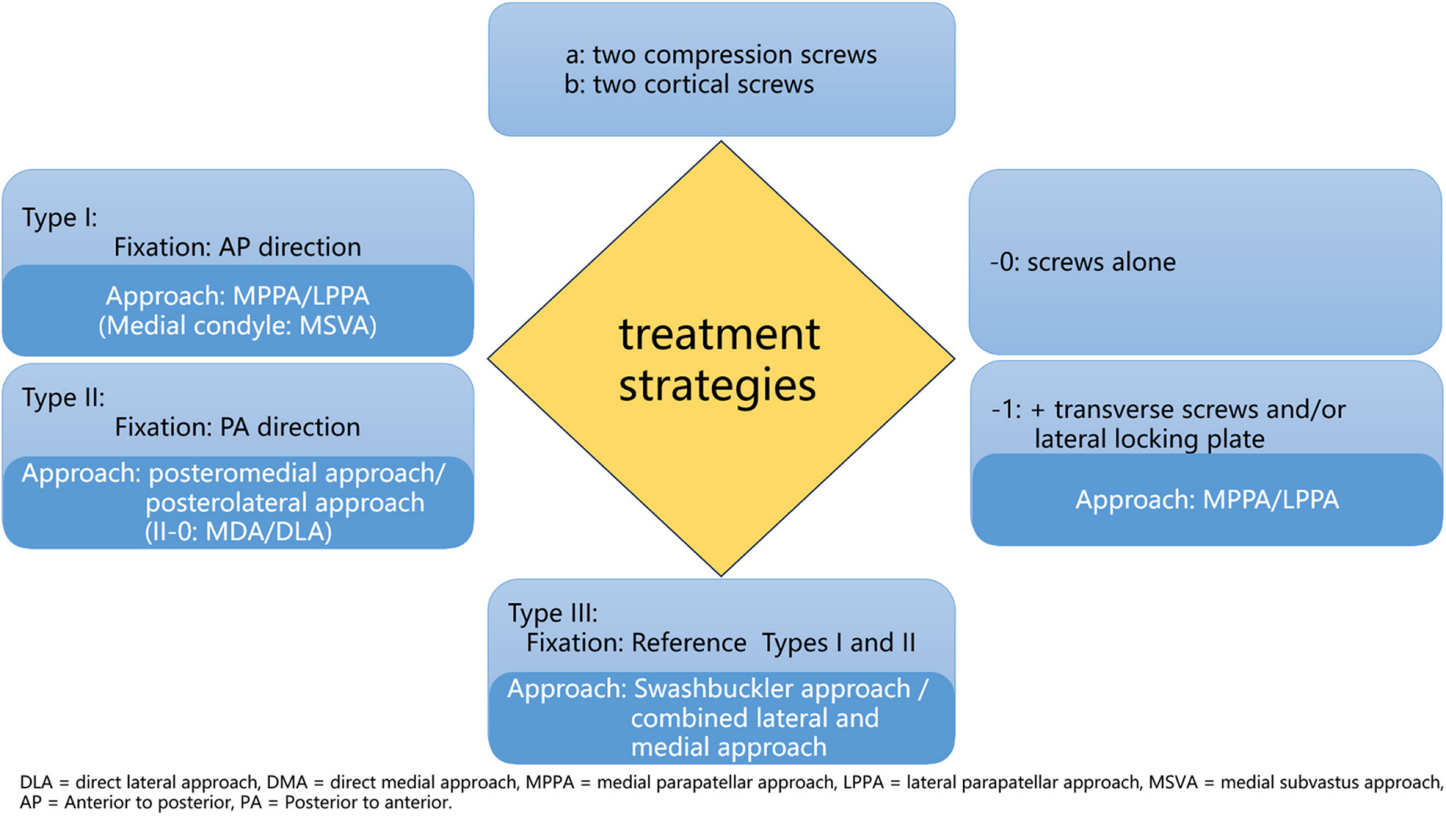
The treatment strategy combination flowchart.

Type IV is a particular type of fracture, such as anterior Hoffa fracture and osteochondral fracture. These fractures are extremely rare, and the treatment experience is somewhat limited. Anterior Hoffa fracture of the medial condyle can be treated through the MSVA, while the anterior Hoffa fracture of the lateral condyle is treated through the LPPA. The combination of arthroscopically assisted reduction and percutaneous screw fixation for the anterior Hoffa fracture is also a good option; however, it demands higher technical proficiency and ample experience (30, 31). In the case of osteochondral fracture, the medial condyle is approached via the DMA, whereas the lateral condyle is approached via the Posterolateral approach (PLA). Owing to the involvement of the cartilage surface and the small size of the fracture fragment, it is recommended to utilize headless compression screws or bioabsorbable screws for fixation (23, 32–34). Considering that perpendicular screws offer the optimal mechanical treatment, numerous scholars have underlined the significance of perpendicular screw compression fixation for fractures (25, 26, 35–37), we suggest applying screw compression technology for simple splitting fractures or fractures without comminution in the screw channel area. The optimal treatment strategies for each subtype are described in Table 7. We also constructed the corresponding treatment strategy figure to facilitate understanding (Figure 1). Clinicians can identify appropriate suffixes in the figure according to subtype classifications to formulate treatment strategies. Type IV represents a distinct and rare fracture subtype requiring separate consideration; therefore, it was excluded from the figure.

**Table 7:** Optimal treatment strategies for each subtype.

Classification	Approach		Internal fixation methods
	Medial condyle	Lateral condyle	
Ia-0	MPPA/MSVA	LPPA	Two AP compression screws
Ia-1	MPPA/MSVA	LPPA	Two AP compression screws + transverse screws and/or lateral locking plate
Ib-0	MPPA/MSVA	LPPA/DLA	Two AP cortical screws
Ib-1	MPPA/MSVA/Swashbuckler approach	LPPA/Swashbuckler approach	Two AP cortical screws + lateral locking plate
IIa-0	DMA/posteromedial approach	DLA/posterolateral approach	Two PA compression screws
IIa-1	MPPA/posteromedial approach	LPPA/posterolateral approach	Two PA compression screws + transverse screws and/or lateral locking plate
IIb-0	DMA/posteromedial approach	DLA/posterolateral approach	Two PA cortical screws
IIb-1	MPPA/MSVA	LPPA	Two PA cortical screws + lateral locking plate
IIIa-0	Swashbuckler approach/combined lateral and medial approach		Reference to type Ia-0/IIa-0
IIIa-1	Swashbuckler approach/combined lateral and medial approach		Reference to type Ia-1/IIa-1
IIIb-0	Swashbuckler approach/combined lateral and medial approach		reference to type Ib-0/IIb-0
IIIb-1	Swashbuckler approach/combined lateral and medial approach		reference to type Ib-1/IIb-1
IV: anterior Hoffa fracture	MSVA/arthroscopy	LPPA/arthroscopy	Headless compression screws/bioabsorbable screws
IV: Osteochondral fracture	DMA	PLA	headless compression screws/bioabsorbable screws

DLA, direct lateral approach; DMA, direct medial approach; MPPA, medial parapatellar approach; LPPA, lateral parapatellar approach; MSVA, medial subvastus approach; PLA, posterolateral approach; AP, anterior to posterior; PA, posterior to anterior.

Given the low incidence rate of Hoffa fractures, detailed preoperative planning based on the morphological characteristics of the fractures is of significant clinical value for orthopedic surgeons with limited experience. The novel CT-based classification system helps to shorten the experience-dependent learning curve of Hoffa fractures by systematically analyzing fracture characteristics and providing evidence-based surgical strategies. Precise preoperative planning enables mitigation of surgical complexity and reduction in intraoperative fluoroscopy frequency, thereby decreasing procedural duration and enhancing the operation's efficiency and safety. Standardized internal fixation protocols are conducive to establishing the biological environment of fracture healing and rapid postoperative rehabilitation of patients.

The proposed classification system demonstrates good inter-observer agreement and excellent intra-observer consistency, confirming its robust reproducibility and criterion validity. During variability tests, observers tended to confuse the new classification type Ib with type IIb when evaluating certain comminuted fractures (equivalent to Letenneur type I and IIa comminuted Hoffa fractures), posing challenges for therapeutic decision-making alignment. Given comparable biomechanical stability between AP and PA screw fixation for Letenneur type I and IIa Hoffa fractures (4, 6, 8), with the added advantages of technical accessibility and low neurovascular injury risks for AP screw, we recommend adopting the novel classification type Ib for surgical planning in these cases.

Our study, based on the Letenneur classification and recent CT imaging features of Hoffa fracture, combined with previous biomechanical studies and case reports, proposed a novel classification with high clinical application value. In clinical diagnosis and treatment, the following factors exert a decisive impact on the treatment strategy for Hoffa fracture: the size of the fracture fragment, which is the critical factor (25); whether comminuted fracture or not (38); whether collapsed articular surface or not (24, 39); and whether comminuted with intercondylar or supracondylar fracture (16). The classification system proposed in this study integrates these factors as the basis for classification, being close to clinical practice and offer more valuable reference for selecting surgical approaches and fixation methods. Furthermore, the novel classification system covers all currently recognized Hoffa fracture types, including relatively rare bicondylar fractures, as well as rare anterior Hoffa fracture and osteochondral fracture, making it universally applicable. Its four broad types differ significantly in anatomical structure, biomechanics, imaging, and treatment methods, facilitating better distinction, comprehension, and mastery. At the same time, we also consider previous biomechanical studies, making the novel classification system more scientific. Through the reliability verification between inter- and intra-observer, it was manifested to possess satisfactory reliability. Finally, we also propose corresponding surgical approaches and fixation methods for each subtype, offering a reference for the formulation of clinical treatment strategies.

Although the proposed classification system in this study establishes a structured framework for clinical decision-making, its limitations require objective acknowledgment. First, as a retrospective exploratory investigation constrained by the low incidence and high rate of missed diagnoses associated with Hoffa fractures, our study had a relatively small sample size and incomplete data, which may compromise statistical power. Prospective multicenter studies with expanded samples (including minor populations) are needed for further validation. Second, several critical confounding factors were not systematically evaluated: patient age-related variations (e.g., osteoporosis impacting fixation choices), diverse injury mechanisms (e.g., high-energy trauma leading to complex fractures that influence surgical approach choices), comorbidities (e.g., diabetes impairing bone healing), and surgeon experience (e.g., junior surgeons prefer for anterior approaches) may collectively influence therapeutic decisions. While these factors extend beyond the primary scope of this classification validation phase, their clinical interactions necessitate comprehensive integration into individualized management strategies. Moreover, the current classification system, derived exclusively from adult data, remains unvalidated for minors' applicability and long-term prognostic efficacy. This system should be regarded as a dynamic clinical tool, requiring flexible, individualized adaptation to clinical contexts. We recommended cautious interpretation regarding clinical treatment strategy guidance intensity within the novel classification system. Future research will establish multicenter prospective cohorts to quantify confounding factor weights through multivariate modeling and develop prognostic evaluation frameworks to enhance clinical translation.

## 5 Conclusion

The CT-based novel classification method proposed in this study, distinct from existing classification systems, integrates fracture fragment size, fracture-end comminution, articular surface collapse, and intercondylar/supracondylar involvement, comprehensively encompassing all fracture types. While presenting a certain complexity, it enables a more comprehensive characterization of fracture features. Furthermore, this classification system helps to enhance standardized clinical decision-making and prognosis optimization by providing evidence-based treatment strategies. It demonstrates good inter-observer agreement and excellent intra-observer consistency, confirming its robust reproducibility and criterion validity. Future multicenter prospective studies are still necessary to validate this classification system.

## Data Availability

The original contributions presented in the study are included in the article/Supplementary Material, further inquiries can be directed to the corresponding author.
